# Moving beyond gene expression: identification of lung-disease-associated novel transcripts and alternative splicing by RNA sequencing

**DOI:** 10.1186/1753-6561-6-S6-P4

**Published:** 2012-10-01

**Authors:** John Brothers, Rebecca Kusko, Gang Liu, Lingqi Luo, Brenda Juan Guardela, John Tedrow, Yuriy Alekesyev, Ivana V Yang, Mick Correll, Mark Geraci, John Quackenbush, Frank Sciurba, Paola Sebastiani, Marc Lenburg, Naftali Kaminski, David A Schwartz, Avrum Spira, Jennifer Beane

**Affiliations:** 1 Bioinformatics Program, Boston University, Boston, MA 02115, USA; 2 Genetics and Genomics Graduate Program, Boston University School of Medicine, Boston, MA 02118, USA; 3 Section of Computational Biomedicine, Department of Medicine Boston University School of Medicine, Boston, MA 02118, USA; 4 Simmons Center for Interstitial Lung Disease and Department of Medicine, University of Pittsburgh Medical Center, Pittsburgh, PA 15213, USA; 5 Department of Pathology and Laboratory Medicine, Boston University School of Medicine, Boston, MA 02118, USA; 6 Center for Genes, Environment and Health and Department of Medicine, National Jewish Health, Denver, CO 80206, USA; 7 Dana-Farber Cancer Institute and Harvard School of Public Health, Boston, MA 02115, USA; 8 Department of Medicine, University of Colorado School of Medicine, Aurora, CO 80045, USA

## Background

Chronic lung diseases affect a significant portion of the population, and the incidences of chronic obstructive pulmonary disease (COPD)/emphysema and idiopathic pulmonary fibrosis (IPF) are increasing. COPD is the fourth leading cause of death in the USA and the incidence of IPF has doubled over the past decade. Identification of novel transcripts and transcript isoforms (alternative splicing patterns) associated with these diseases may help us better understand their molecular pathogenesis, and identify both novel disease-specific biomarkers and therapeutic targets.

## Materials and methods

Using lung tissue sections from the NHLBI Lung Tissue Research Consortium, we sequenced the mRNA (75 or 99 nt paired-end sequencing; Illumina GAIIx or HiSeq) from 145 lung tissue samples that were subsequently split into an initial training cohort of 89 samples and an independent filtering set of 56 samples. Genome-guided transcriptome reconstruction using Cufflinks was performed on the training and independent filtering set. A final conservatively filtered assembly was created by requiring complete overlap of all transcripts present for a gene in the two assemblies. Next, the algorithm MISO was used to quantify isoform proportions for known and novel transcripts found in each gene. These were modeled as a function of the disease state, isoform and the interaction between disease state and isoform to identify disease-associated differentially spliced genes.

## Results

The filtered transcriptome assembly (overlap set) is more similar to known genes (based on comparisons with Ensembl) than the initial training and independent filtering set. A set of 38 novel gene candidates were selected based on gene structure parameters computed from Ensembl annotation. Differential expression (DE) analysis was performed, and five of the candidate genes were DE in emphysema and eight in IPF (*P*<0.01) compared with control. Three of these candidate genes were DE in both diseases. Several examples of disease-associated differential splicing were also identified. These new disease-associated isoforms are being further investigated to identify their biological function and relevance to COPD and IPF.

## Conclusions

RNA-Seq of a large number of lung tissue samples has allowed us to identify novel disease-associated genes and alternative splicing patterns that may contribute to our understanding of the pathogenesis of IPF and COPD.

